# Emulsion and Surface-Active Properties of Fish Solubles Based on Direct Extraction and after Hydrolysis of Atlantic Cod and Atlantic Salmon Backbones

**DOI:** 10.3390/foods10010038

**Published:** 2020-12-25

**Authors:** Silje Steinsholm, Åge Oterhals, Jarl Underhaug, Tone Aspevik

**Affiliations:** 1 Nofima AS, P.O. Box 1425 Oasen, N-5844 Bergen, Norway; aage.oterhals@nofima.no (Å.O.); tone.aspevik@nofima.no (T.A.); 2 Department of Chemistry, University of Bergen, Allégaten 41, N-5020 Bergen, Norway; jarl.underhaug@uib.no

**Keywords:** enzymatic protein hydrolysates, emulsion activity, critical micelle concentration, fish by-products, emulsion stability

## Abstract

The focus on natural foods and “clean” labeled products is increasing and encourages development of new biobased ingredients. Fish solubles derived from downstream processing of side stream materials in the fish filleting industries have potential as emulsifiers based on their surface-active and emulsion stabilizing properties. The aim of this study was to evaluate and compare emulsion properties and critical micelle concentration (CMC) of direct protein extracts and protein hydrolysates based on fish backbones, and to identify associations between molecular weight distribution and process yield with the studied physicochemical properties. Protein extracts and enzymatic protein hydrolysates were produced based on two raw materials (cod and salmon backbones), two enzymes with different proteolytic specificity, and varying hydrolysis time. Emulsion activity index (EAI), emulsion stability index (ESI) and CMC were measured and compared with casein as a reference to protein-based emulsifiers. Protein hydrolysis was found to have negative impact on EAI and CMC, likely due to generation of small peptides disrupting the amphiphilic balance. The direct protein extracts had comparable EAI with casein, but the latter had superior ESI values. Protein hydrolysates with acceptable EAI could only be obtained at the expense of product yield. The study emphasizes the complexity of physicochemical properties of protein hydrolysates and discusses the challenges of achieving both good surface-active properties and high product yield.

## 1. Introduction

Emulsifiers are important ingredients in a variety of formulated food products containing two immiscible phases, such as mayonnaise, spreads, and salad dressings [1]. Their surface-activity reduces the interfacial tension between the phases and promotes the formation of stable emulsions. Present consumer attention and increasing preference for natural products and “clean” labeled food products is encouraging the development of new natural surface-active biobased ingredients [2,3]. Food-approved emulsifiers include proteins, polysaccharides, phospholipids, and synthetic surfactants [1,4]. Fish-based peptides may be a coming alternative. Given adequate surface-activity, this type of emulsifier will also add to the nutritional value while exerting a function in food formulations.

Emulsions in foods are often in the form of oil-in-water (O/W), where small droplets of lipids are distributed in a continuous aqueous phase, or water-in-oil (W/O), where oil is the continuous phase [1]. The formed emulsions are thermodynamically unstable and require the presence of an emulsifier for stabilization through reduction of surface-tension and prevention of droplet aggregation and coalescence. The amphiphilic nature of proteins facilitates adsorption at the interphase between polar and non-polar environments, after which they will reorient in a manner maximizing the contact between hydrophilic areas with the oil, while repelling other emulsion droplets [5,6]. The adsorption rate of native proteins to an interphase varies depending on the protein, but often the net charge of the proteins will not provide repulsion between droplets, causing aggregation [1]. Partial hydrolysis may improve the physicochemical properties due to exposure of hydrophobic moieties, improving the electrostatic balance, and increase the solubility and flexibility of the peptides compared to the intact protein [7,8].

Several studies have addressed the physicochemical properties of protein hydrolysates [9,10,11,12,13,14,15,16,17,18,19,20,21], where influences of both hydrolysis time and choice of enzyme have been assessed. The degree of hydrolysis determines the reduction in peptide molecular weight, while enzyme specificity influences the balance between hydrophilic and hydrophobic regions, both imperative for peptide surface-activity. Enzymatic protein hydrolysates of side stream products from the fish filleting industry, such as heads, backbones, and trimmings, have been proposed as a source of emulsifiers for food formulations [8]. This represents a possibility for valorization of side streams and increasing the yield of water-soluble protein, while simultaneously improving the functionality of the native proteins [22]. Hydrolysis of herring protein has been shown to improve emulsion activity and stability [23], and sardine protein hydrolysates showed better emulsion properties compared with sodium caseinate [24]. However, hydrolysis of the common emulsifiers casein and whey have not always been found to improve physicochemical properties (emulsion capacity and stability), having emulsifying abilities either inferior or comparable to that of the native proteins [24]. In general, large peptides (>2–4 kDa) are essential for proper functionality, and it has been suggested that peptides should contain more than 20 amino acids to exhibit good emulsifying capability [16]. In addition, peptide–peptide interactions are particularly important [5,10,16].

Several approaches can be used in the determination of surface-activity and emulsion properties of proteins and peptides. Emulsion activity (emulsion activity index; EAI) determines the obtainable interfacial area between oil and water per unit weight of protein or product, and emulsion stabilizing ability (emulsion stability index; ESI) indicates the emulsifying effect over time [25]. Critical micelle concentration (CMC) indicates the minimum concentration of a product needed for maximum reduction of the surface-tension and can be assessed by different methods based on fluorescence, conductivity, surface-tension, or ^1^H nuclear magnetic resonance (NMR) [26,27]. High CMC values imply poor surface-activity, i.e., a high concentration of the given surfactant is needed to reduce the surface-tension [10,27]. Measurements of properties related to emulsion capabilities of protein hydrolysates are challenging to standardize. Pearce and Kinsella [25] showed that EAI results are dependent on assay variations, such as homogenization factors and protein concentration, and the latter was confirmed by Nalinanon et al. [17]. This makes interstudy comparisons difficult and may add to the contradictory results from previous emulsion studies of protein hydrolysates.

There is a lack of knowledge on the effect of raw material and process variables on properties related to surface-activity of fish solubles. The aims of this study were (1) to evaluate and compare physicochemical properties (i.e., emulsion activity (EAI), stability index (ESI) and CMC) based on direct extraction and enzymatic hydrolysis of salmon and cod backbones, and (2) to assess the association between peptide molecular weight distribution, physicochemical properties, and process yields.

## 2. Materials and Methods

### 2.1. Materials

Atlantic salmon (*Salmo salar*) and cod (*Gadus morhua*) backbones were kindly provided by Sotra Fiskeindustri AS (Glesvær, Norway) and Halstensen Granit AS (Bekkjarvik Norway), respectively. The raw materials were milled on a Comitrol 1700 (Urschel laboratories, Chesterton, IN, USA), vacuum packed, and stored at −20 ℃ until use. The applied enzymes were Bromelain BR1200 (EC 3.4.22.32, Enzybel, Waterloo, Belgium) and FoodPro PNL (EC 3.4.24.28, DuPont, Wilmington, DE, USA). Refined rapeseed oil was purchased at a local supermarket (Rema 1000 store brand, Kjerreidviken, Norway). Peptide standards were purchased from Sigma-Aldrich (Oslo, Norway) except lysozyme (Fluka biochemicals, Buchs, Switzerland) and Alberta standards (Alberta Peptide Institute, Department of Biochemistry, University of Alberta, Edmonton, AB, Canada). Technical grade Tween 20 (VWR, Oslo, Norway) and bovine casein (Sigma, Oslo, Norway) were applied in the emulsion assay. All other chemicals were analytical or food grade.

### 2.2. Chemical Analyses

Analysis of nitrogen (N) was performed by the Kjeldahl method [28] and the crude protein level determined based on substrate specific N-to-protein conversion factor [29]. Amino acid composition was quantified by High performance liquid chromatography (HPLC) using fluorescence detection with excitation/emission at 250/395 nm. Proteins were hydrolyzed to free amino acids with 6N HCl and amino acids derivatized with 6-aminoquinolyl-*N*-hydroxysuccinimidyl carbamate before passing through the HPLC column (Waters Accq Tag 3.9 × 150 mm, Milford, MA, USA) and detector [30]. Asparagine and glutamine were estimated based on the release of ammonia in the HCl digest compared to a neutral control sample [31]. Released ammonia was quantified by the method of Conway and Byrne [32]. Analysis of molecular weight distribution (MWD) was performed by HPLC (1260 series HPLC, Agilent Technologies, Santa Clara, CA, USA) using size exclusion chromatography [33], as described by Oterhals and Samulesen [34]. All chemical analyses were performed in duplicate with predetermined allowances for replicate variation.

### 2.3. Enzymatic Protein Hydrolysis

Raw materials were thawed overnight at 4 °C. The raw material was mixed with purified water (1:1) and transferred to a Distek Model 2500 Dissolution System (Distek Inc., North Brunswick, NJ, USA). The slurry was heated to 50 °C at continuous stirring (70 rpm) before adding 10 U enzyme per gram protein [35]. The proteolytic reactions were terminated after 5, 10, 30 or 60 min by heating to >90 °C in a microwave oven (Menumaster commercial, Cedar Rapids, IA, USA) for a minimum 10 min. The slurry was cooled to <40 °C in a water bath before phase separation by centrifugation at 15,000× *g* for 20 min (Sorvall, LYNX 6000, Thermo Scientific, Waltham, MA, USA). Direct protein extracts by thermal coagulation were produced with the same method, with the exception of enzyme addition, of both raw materials. The water phase was filtered through a Seitz-T2600 filter (Mall Corporation, East Hills, NY, USA) to remove larger particles and thereafter subjected to 0.1 µm cross flow membrane filtration (Centramate 500S Tangential Flow Filtration System, Pall, Port Washington, NY, USA) to remove remnant fine particles and fat. The final hydrolysates were stored at −20℃ until further use.

Protein recovery (PR) was determined based on protein content in the filtered hydrolysate compared to that in the raw material:

(1) $$\mathrm{PR}=\frac{\mathrm{Protein}\;\mathrm{in}\;\mathrm{the}\;\mathrm{hydrolysate}\times g\;\mathrm{hydrolysate}}{\mathrm{Protein}\;\mathrm{in}\;\mathrm{the}\;\mathrm{raw}\;\mathrm{material}\times g\;\mathrm{raw}\;\mathrm{material}}\times 100\%$$

volume of collected hydrolysate after membrane filtration was corrected for the remaining dead volume in the Centramate filter apparatus.

### 2.4. Determination of Critical Micelle Concentration by NMR Spectroscopy

All samples were diluted 3:4 with 400 mM sodium phosphate buffer (pH 6.5) containing 15% D_2_O and 2,2-dimethyl-2-silapentane-5-sulfonate (DSS). The pH was adjusted to 6.5 with 0.1 M HCl. Dilution series of 10 samples were prepared from all hydrolysates with the final concentration of 100 mM sodium phosphate buffer and 7.5% D_2_O. A volume of 600 µL was transferred to 5 mm NMR tubes. ^1^H spectra were acquired at 300 K with a Bruker AVANCE NEO ultrashielded 600 MHz spectrometer with cryoprobe using Bruker pulse program zgesgppe (Karlsruhe, Germany). Acquisition parameters were set to four dummy scans, 32 real scans, 1 s relaxation delay, 32k time-domain points, and spectral width of 11.9 ppm. The NMR spectra were processed using TopSpin (v. 4.0.4, Bruker BioSpin, Karlsruhe, Germany). Exponential line broadening of 0.5 Hz was applied prior to Fourier transformation, and the chemical shifts were referenced to DSS.

The CMC was estimated by plotting the ^1^H shift of the lactate methyl group as a function of log protein concentration [10]. The methyl resonance of lactate was visible around 1.3 ppm for all samples. Separate linear trend lines were drawn for the lag phase and the exponential phase in the plot. The data points used for the lag phase tangent were adjusted to obtain best fit by removing points in the transition between lag and exponential phase. The intercept of these lines indicated the start of protein aggregation and thus the critical micelle concentration. Propagation of uncertainty assuming independent variables indicated standard measurement error < ± 0.2 based on the fitted regression lines and standard error for the coefficient provided by the LINEST function in Microsoft Excel (v. 2013). Aspevik et al. [10] estimated the standard deviation for the used protocol to be 0.5 g/L.

### 2.5. Emulsion Properties

Emulsion activity index (EAI) and emulsion stability index (ESI) were determined based on the method by Pearce and Kinsella [25] and modified by Liceaga-Gesualdo and Li-Chan [23]. The hydrolysates were diluted to 1.0% protein in purified water and pH adjusted to 6.5 with 0.1 M HCl. Tween 20 was used as control to ensure method repeatability, and casein (1% solubilized in 50 mM potassium phosphate) as a reference to commercial emulsifiers. Emulsions were made by adding 2 mL of rapeseed oil to 6 mL of standardized hydrolysate and homogenized (T 10 basic Ultra-Turrax, Ika, Staufen, Germany) at 16,000 rpm for 1 min in a glass container. Emulsions were made in triplicate. Immediately after homogenization, 25 µl was collected from the bottom of the mixture, added to 5 mL 0.1% sodium dodecyl sulfate (SDS) and the mixture inverted two times before measurement of EAI. Sample collection was repeated after 10 and 30 min for determination of ESI. Absorbance was measured at 500 nm and EAI was calculated based on the following equation:

(2) $$\mathrm{EAI}\;\left(m^{2}{\left(g\;\mathrm{protein}\right)}^{-1}\right)=\frac{2\times \frac{2.303A}{l}\times \mathrm{df}}{\varphi \times c}$$

where A = absorbance measured at 0, 10 or 30 min; l = path length of cuvette in m; df = dilution factor (200); *φ* = oil phase volume of total mixture volume; c = weight of protein per unit volume of aqueous sample before emulsion. ESI was calculated based on the percent EAI remaining after a defined stagnant standing period:

(3) $$\mathrm{ESI}\left(\%\right)=100-\left(\frac{\mathrm{EAI}-{\mathrm{EAI}}_{t}}{\mathrm{EAI}}\times 100\right)$$

where EAI_t_ = EAI value of samples collected after 10 or 30 min.

### 2.6. Statistics

Analysis of variance (ANOVA) was performed using Minitab (v. 19.2, Pennsylvania State University, PA, USA). One-way ANOVA was used to determine significant product differences of EAI and ESI results. Two-way ANOVA determined significant parameters. Hydrolysates were used to model effect of raw material, enzyme, and hydrolysis time. Tukey’s pairwise comparison was used when significance (*p* < 0.05) was found.

Principal component analysis (PCA) was used to evaluate relationships between EAI, ESI, and MWD using Unscrambler (v10.4.1, Camo, Oslo, Norway). All data were unit variance scaled and centered before analysis.

## 3. Results and Discussion

### 3.1. Raw Material and Hydrolysate Composition

Salmon and cod backbones showed similar amino acid compositions (Table 1), with slightly higher levels in the cod raw material, reflecting higher protein concentration in the latter substrate. Both substrates contained high levels of glycine, proline, and hydroxyproline, ascribed to the high proportion of bones and connective tissue protein. N-to-protein conversion factors (f_N_) for cod and salmon backbones were calculated to 5.5 and 5.2, respectively, in agreement with previous findings for pure muscle proteins from the two species [35], and illustrated the deviance of fish raw material from the commonly used factor of 6.25. The use of substrate specific conversion factors facilitated a more accurate quantification of protein content, and thus enzyme addition on protein basis and standardization of hydrolysis conditions in studies applying different protein sources [30].

The enzymes were added based on similar activity-to-protein ratio [31], and the resulting peptide molecular weight distribution (MWD; Table 2) showed an increase in smaller peptides as the hydrolysis progressed. Furthermore, levels of molecules <0.2 kDa were higher in products based on FoodPro PNL compared to the equivalent Bromelain hydrolysate, indicating some exopeptidase activity in the former enzyme [31]. In general, hydrolysates based on cod backbone contained a larger proportion of peptides, >1 kDa, compared with salmon. For both raw materials, the PR increased with prolonged hydrolysis time, with Bromelain giving slightly higher levels compared with FoodPro PNL (Table 3). This is possibly explained by the broad specificity of Bromelain and efficiency on connective tissue proteins [36]. As expected, the direct protein extracts contained mostly large peptides, >10 kDa, and small molecules, <0.2 kDa, characteristic for a product based on direct thermal coagulation and separation [10,34].

All products were purified by microfiltration to eliminate suspended solids and residual lipids with possible bias effects in the surface-activity assays. The protein recoveries (Table 3) were lower than observed in earlier studies without a microfiltration step [10], particularly for the direct extracts (PR = 6–7% compared to expected approximate 20%). This confirms a partial retention of large proteins and peptide fragments by microfiltration, as earlier reported [37]. The shift in MWD toward smaller peptides probably influenced the surface-active properties of the respective products; however, effects of membrane filtration were outside the scope of this study.

### 3.2. Associations between MWD and Physicochemical Properties

**Figure 1:**
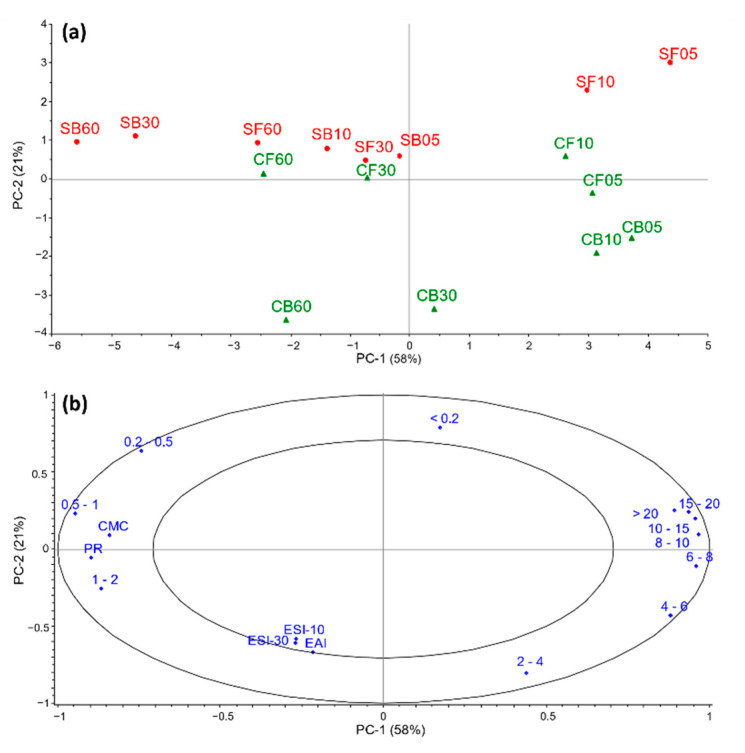
Principal component analysis score plot (**a**) shows similarities and differences between salmon (S) and cod (C) backbone hydrolysates made with FoodPro PNL (F) and Bromelain (B) for 5, 10, 30 and 60 min. The correlation loading plot (**b**) illustrates associations between molecular weight distribution (kDa), protein recovery (PR), critical micelle concentration (CMC), emulsion stability after 10 min (ESI-10), emulsion stability after 30 min (ESI-30), and emulsion activity index (EAI). The two ellipses represent 50% and 100% of explained variance.

Principal component analysis (PCA; Figure 1) was used to evaluate associations between EAI, ESI, CMC, PR, and MWD of the hydrolysates. Data from the direct protein extracts were excluded due to the deviant MWD and PR compared with the hydrolysates (Table 2 and Table 3), adding too much leverage to the model and dominating the variation of the two variables. Based on the score plot, hydrolysates with similar or different properties could be identified. Two principal components (PCs) were found to be relevant for the interpretation of results. The first and second PCs explained 58% and 21%, respectively.

In the score plot (Figure 1a), PC-1 mainly explains the effect of hydrolysis time, while the raw material variation is explained by PC-2. The correlation loading plot (Figure 1b) mostly shows a product separation based on CMC, PR, and MWD. High values for the two former variables and small molecules of 0.5–2 kDa were associated and negatively correlated with molecules of 4–>20 kDa, in agreement with Aspevik et al. [10]. The emulsion responses were less than 50% explained by the model, thus interpretation should be done with care. The loading plot shows no positive correlations between emulsion properties and specific MW groups. This was also the case in studies on salmon muscle protein hydrolysates [15], and whey and casein protein hydrolysates [24]. The results indicate that extended hydrolysis is detrimental to surface-activity, expressed by CMC. The MWD (Table 2) and considerably lower CMC values (Table 3) for the direct protein extracts supported this conclusion.

### 3.3. Effect of Process Parameters on Emulsion Properties

**Figure 2:**
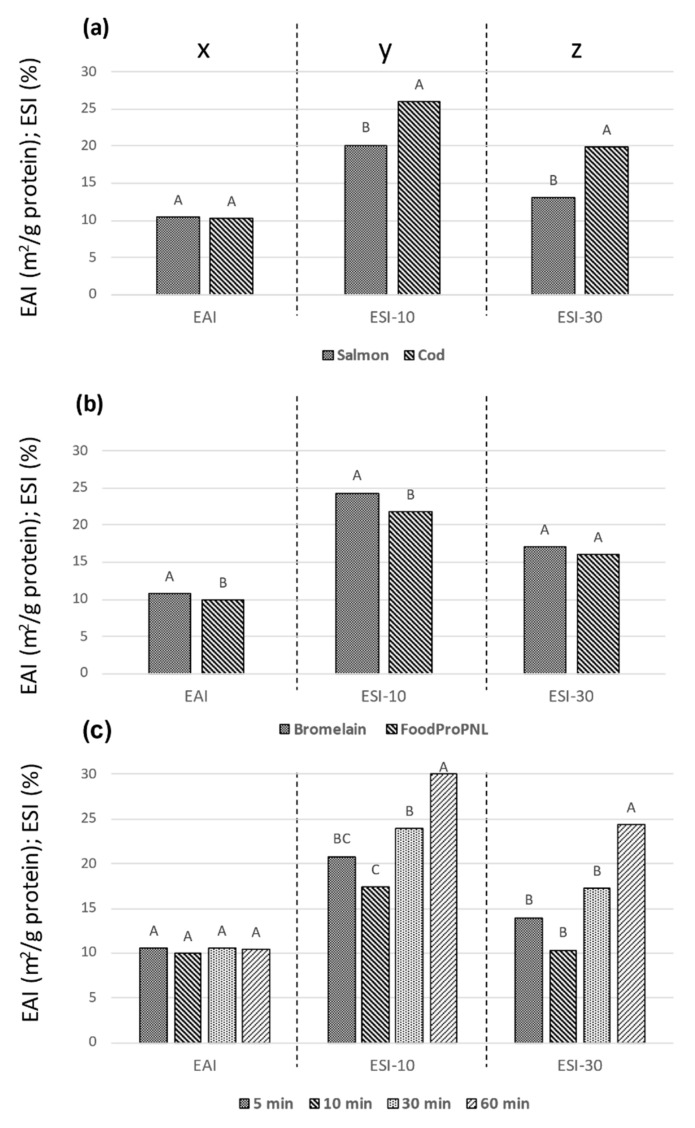
Mean emulsion activity index (EAI) and emulsion stability index (ESI) after 10 and 30 min for the raw materials salmon and cod (**a**), the enzymes FoodPro PNL and Bromelain (**b**), and hydrolysis times (**c**). Different letters indicate statistical effect of the hydrolysis parameter on the emulsion variable. EAI (x), EAI-10 (y) and EAI-30 (z) are separate statistical entities, indicated by the dotted lines.

The EAI of the products (Table 3) showed small differences between the enzymatic hydrolysates, but the direct protein extracts gave the highest values for both cod and salmon. This may be attributed to the higher levels of large peptides (>10 kDa) being sufficiently flexible for effective interfacial surface coverage. Furthermore, a decrease in surface hydrophobicity of the hydrolysates may also add to this observation, as discussed for whey hydrolysates [20]. Negligible differences between the EAI (Table 3) of the hydrolysates were observed, with no clear pattern of differences influenced by raw material, enzyme, or hydrolysis time (Figure 2).

Casein is an excellent emulsifier in milk-based products [6], and the EAI of casein was measured at 16 ± 1 m^2^/g protein, only slightly higher than the direct protein extracts in this study (EAI = 12–13, Table 3). We have found few studies comparing the EAI of fish-based protein hydrolysates with direct protein extraction of the raw material. Contrary to this study, Liceaga-Gesualdo and Li-Chan [23] showed enhanced EAI of herring hydrolysates compared to this type of reference sample. More common is to use a commercial protein emulsifier as reference. Tan et al. [21] found that restricted hydrolysis on catfish gave EAI and ESI comparable to those of soy protein isolate. However, Alves et al. [9] found the EAI of soy proteins to be superior to that of chicken blood hydrolysates and associated this to large interfacial areas of the soy proteins. The interfacial properties of proteins could possibly explain the relatively good results for the casein protein and the direct thermal extracts compared to the hydrolysates in this study. The mentioned studies [6,9,21,23] had assay variations comparable to the current work, such as sample dilution media, protein concentration, and pH, likely influencing the results.

The ESI showed more variation between the samples compared to the EAI (Table 3), with lowest values obtained by the direct protein extracts. The low stability of the two latter emulsions may be due to a combination of electrostatic attraction between unfolded protein and interactions with other small molecules present in the samples [1]. An increase in ESI with prolonged hydrolysis time for the salmon hydrolysates was observed, suggesting that a higher release of small molecules is required for stabilization, compared with shorter hydrolysis time (Figure 2). Other studies have reported a decrease in emulsion stability in hydrolysates based on salmon heads [13], muscle proteins [15], and chicken blood [9] when the degree of hydrolysis is increased. They attributed the reduction in ESI to a reduction in interfacial tension and increased hydrolysis, indicating a loss of emulsion stabilizing properties when the salmon peptides are substantially hydrolyzed.

The ESI values for the cod hydrolysates, on the other hand, were more ambiguous, with low levels at intermediate hydrolysis times. The highest observed levels were, however, similar for both salmon and cod, and the values decreased after 30 min holding time. The ESI values of casein were about two times higher compared with the hydrolysates, with values for ESI-10 = 54 ± 5 and ESI-30 = 40 ± 4, likely due to an appropriate balance of hydrophobic regions [1] and high film viscosity.

ANOVA of the individual enzymatic hydrolysis parameters demonstrated that there were no significant effects of species or hydrolysis time on EAI (Figure 2(ax,cx)), whereas Bromelain gave significantly higher values compared with FoodPro PNL (Figure 2(bx)). The proteases Bromelain and FoodPro PNL (formerly named Protex 7L) were chosen based on studies suggesting good emulsion properties and CMC values in hydrolysates based on these enzymes [10,12]. The observed difference, although small, was in agreement with a study on tilapia hydrolysates [12], where Bromelain gave superior emulsion properties of the four proteases tested.

All hydrolysis parameters significantly influenced the ESI-10 levels (Figure 2(ay–cy)).where cod, Bromelain, and 60 min hydrolysis were superior to salmon, FoodPro PNL, and shorter hydrolysis time, respectively. This suggests that smaller peptides may be important for emulsion stability, in agreement with other studies on fish-based substrates [17,18]. On the other hand, there have been studies suggesting a general decrease in emulsion properties of fish-based substrates as the hydrolysis progressed [14,15], but very high protease concentration [14] and different emulsion assays [15] were applied. The PCA-plot (Figure 1) indicates a negative association between MW < 0.2 kD and ESI. Characteristic for the two direct protein extracts is a very high content (60%) of this MW fraction (Table 2) and a low ESI. Combined, this indicates a negative effect of free amino acids on EAI and a positive effect of higher molecular weight peptides in general. No correlations were found between specific peptide fractions and ESI; however, the effect might reflect a low contribution to film viscosity of free amino acids compared to peptides.

Interpretation and comparison between separate studies should be made with caution. The protein concentration applied in EAI assays strongly influences the results [17], along with pH [18] and the equipment used [25]. These factors have been taken into consideration when assessing similarities with other studies, but the challenges emphasize the need for more standardized assay methodology. The process of emulsion formation and stabilization is complex with many influencing factors [5], especially peptide–peptide interactions [16]. Furthermore, the relatively low ESI of the direct extracts (Table 3) shows that the formation and stability of emulsions cannot be seen as co-dependent responses, as they appear to depend on different peptide properties, in agreement with previous studies [24,38].

### 3.4. Critical Micelle Concentration of Fish Protein Hydrolysates

**Figure 3:**
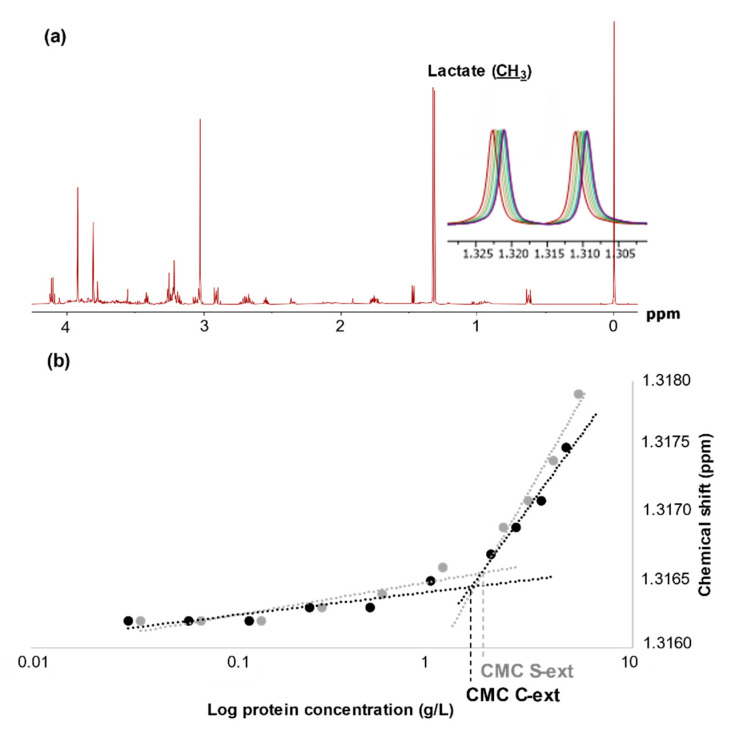
(**a**) ^1^H NMR spectra showing the methyl resonance of lactate around 1.3 ppm, with an insert illustrating the change in chemical shift of lactate with decreasing protein concentration of the salmon protein extract dilution series. (**b**) Determination of the critical micelle concentration (CMC) of the direct protein extracts based on cod (C-ext) and salmon (S-ext).

The use of ^1^H NMR is a well-established method for determination of CMC [10,27]. In this study, the chemical shift of lactate was used to measure changes in the chemical environment due to micelle formation (Figure 3). A full overview of the chemical shifts with the corresponding protein concentration can be found in Table S1. A low CMC is favorable and implies that less of the surfactant is needed to obtain maximum reduction of the surface-tension. The lowest value of CMC was observed for the direct extracts (Table S1) and indicated that the undigested proteins present were flexible enough to exert a better reduction of surface-tension than their peptide moieties. The NMR technology measures a change in the chemical environment; however, it cannot discern if the micelles or aggregates are homogenously distributed, which indicates electrostatic repulsion necessary for emulsifying capabilities. This may add to an explanation of the lack in correlation between CMC and ESI (Figure 1).

An increase in hydrolysis time, and thus reduction in peptide size, gave higher CMC values (Table 3). This was in agreement with a previous observation [10] and likely due to a decrease in the amphiphilic nature of the peptides by extended hydrolysis [6]. The CMC of cod hydrolysates was slightly lower compared with the corresponding salmon hydrolysates and may be explained by the generally higher content of larger peptides in cod-based hydrolysates (Table 2). Furthermore, the CMC values of hydrolysates based on Bromelain were higher than the corresponding FoodPro PNL hydrolysates, probably due to the broad specificity of the former enzyme, leading to more disruption of the hydrophobic areas in the peptides [36]. The obtained CMC values were lower than those reported by Aspevik et al. [10] for salmon heads and backbones, which ranged from 6 g/L in the control sample (without proteolysis) to 11.5 g/L after prolonged hydrolysis with FoodPro PNL. The respective values of the current study are 1.8 and 6.8 g/L. A major difference between the two studies was the use of a 100 kDa [10] and 0.1 µm (this study) membrane filter to remove residual lipids and fine particles in the hydrolysate before measurement of physicochemical properties. A microfiltration step is needed to remove interfering compounds; however, it will also partly remove higher molecular weight peptides with negative impact on CMC. The variation in CMC between the two studies shows that a 100 kDa filter removes more of the high MW molecules required for low CMC values.

The negative correlation between PR and low CMC (Figure 1) suggests that a compromise must be met between high surface-activity and product yield. Although by restricting hydrolysis to a degree where peptide surface-activity is retained, the lower PR may be compensated by introduction of a cascade approach where peptide fractions with different physicochemical properties are obtained after successive hydrolysis steps to improve the overall process yield. Additional studies on droplet size distribution and reduction in interfacial tension may be included to further elucidate the physicochemical properties.

## 4. Conclusions

No associations between CMC and emulsion properties (EAI and ESI) of protein hydrolysates based on cod and salmon backbones were observed. Low CMC, implying good surface-activity, was correlated with peptides > 4kDa and hydrolysates of restricted proteolysis. The lowest CMC and highest EAI values were obtained for products based on direct protein extraction without hydrolysis and reflected a negative effect of hydrolysis on CMC and EAI. The ESI values of the hydrolysates were both increased and reduced compared with direct protein extraction. The process combination of cod, Bromelain, and 60 min of hydrolysis was superior to salmon, Food Pro, and shorter hydrolysis times with respect to this property. The EAI values of direct protein extracts were slightly lower than casein; however, ESI values were less competitive. Hydrolysates showed both lower EAI and ESI values compared to casein. Direct protein extraction gives superior physicochemical properties measured as CMC and EAI; however, it also results in lower ESI and product yield compared with hydrolysis. This is further reinforced by microfiltration to remove residual lipids and fine particles. A cascade approach is suggested as a potential method both to improve product yield and optimize emulsifier properties.

## Figures and Tables

**Table 1:** Amino acid composition in the raw material (g kg^−1^; *n* = 2) and substrate specific nitrogen-to-protein conversion factor data of salmon (*Salmo salar*) and Cod (*Gadus morhua*) backbones.

AA (g/kg) **	Cod Backbone	Salmon Backbone
Alanine	11.0	9.9
Arginine	11.0	9.0
Asparagine *	7.1	6.3
Aspartate	8.9	7.7
Glutamate	16.1	12.0
Glutamine *	7.9	7.0
Glycine	17.0	14.0
Histidine	3.3	3.6
Isoleucine	6.4	5.7
Leucine	12.0	9.6
Lysine	13.0	11.0
Methionine	5.4	4.6
Phenylalanine	5.9	5.2
Proline	8.8	7.8
Serine	8.6	6.5
Threonine	6.9	6.2
Tyrosine	6.9	4.0
Valine	7.4	6.7
Hydroxyproline	3.8	3.5
Nitrogen	27	25
NH3 (acid digest)	1.8	1.6
Total AA	167.4	140.3
Total *n*	27.0	25.0
f_N ***_	5.5	5.2

* Calculated based on released NH_3_, and assuming 1:1 ratio of released NH_3_ between Asp and Glu [29]. ** Amino acid. *** Nitrogen-to-protein conversion factor.

**Table 2:** Apparent molecular weight distribution (MWD; kDa) and average amino acid units in the molecular size of direct protein extracts (-ext) and hydrolysates made from cod (C) and salmon (S) backbones with FoodPro PNL (F) and Bromelain (B) for 5, 10, 30 and 60 min.

MWD (%)	C-ext	CF05	CF10	CF30	CF60	CB05	CB10	CB30	CB60	S-ext	SF05	SF10	SF30	SF60	SB05	SB10	SB30	SB60	AA ^*^
>20	8.9	1.3	1.2	0.4	0.2	1.3	1.4	0.8	0.3	4.9	2.6	1.5	0.4	0.1	1.1	0.8	0.1	<0.1	>198
15–20	3.9	1.1	0.9	0.3	0.1	1.0	0.9	0.5	0.2	2.2	1.7	1.1	0.4	0.1	0.6	0.4	0.1	<0.1	138
10–15	5.7	2.9	2.7	1.1	0.5	3.0	2.6	1.3	0.5	3.1	4.1	3.1	1.3	0.4	1.2	0.8	0.2	<0.1	99
8–10	3.4	3.0	2.9	1.5	0.8	3.5	3.2	1.8	0.8	1.7	4.0	3.4	1.9	0.9	1.0	0.7	0.2	0.1	71
6–8	4.0	6.0	6.1	3.9	2.5	7.8	7.4	5.1	2.8	2.2	7.0	6.5	4.5	2.7	2.5	2.0	0.6	0.3	55
4–6	3.8	12.0	12.6	9.7	7.2	15.9	16.0	13.7	10.0	2.4	11.6	11.2	8.9	6.7	7.6	6.6	2.9	1.7	32
2–4	3.7	21.4	23.0	22.8	21.2	26.4	27.9	30.0	29.3	2.5	18.6	20.0	20.0	18.2	23.3	23.0	16.0	12.0	20
1–2	1.9	16.1	17.2	20.2	21.4	14.6	16.2	20.7	25.0	1.3	12.3	14.0	16.9	18.4	22.2	23.7	25.7	24.5	12
0.5–1	1.2	10.0	11.0	14.9	17.5	6.2	6.9	9.7	13.3	0.8	7.7	9.2	13.0	16.0	12.8	14.3	21.1	24.2	5.9
0.2–0.5	3.3	6.6	7.1	10.3	13.0	3.3	3.4	4.4	6.0	18.4	10.3	11.0	13.6	16.4	10.7	11.5	16.5	20.0	2.8
<0.2	60.3	19.7	15.4	14.8	15.7	17.0	14.1	12.1	11.9	60.7	20.2	19.1	19.1	20.1	16.9	16.4	16.4	17.2	1.0

* Estimated average number of amino acid units in the molecular size group based on weighted average MW [30].

**Table 3:** Measured emulsion activity index (EAI *) and stability index after 10 and 30 min (ESI-10/30 *), critical micelle concentration (CMC **) and protein recovery (PR) of hydrolysates and direct protein extracts (-ext) based on cod (C) and salmon (S) backbones with FoodPro PNL (F) and Bromelain (B) for 5, 10, 30 and 60 min of hydrolysis.

	EAI (m^2^/g)	ESI-10	ESI-30	CMC (g/l)	PR (%)
C-ext	13 ± 0.9 ^a^	21 ± 1.9 ^cde^	16 ± 3.0 ^de^	1.6	6.2
CF05	11 ± 1.2 ^bcd^	33 ± 2.7 ^a^	25 ± 0.6 ^ab^	3.7	18.1
CF10	9.0 ± 0.2 ^cd^	18 ± 1.9 ^def^	9 ± 0.6 ^efg^	4.8	23.4
CF30	11 ± 0.7 ^bcd^	14 ± 2.1 ^ef^	7 ± 1.3 ^fgh^	6.0	31.4
CF60	9.0 ± 0.2 ^d^	27 ± 0.5 ^abcd^	22 ± 2.9 ^abc^	6.2	35.6
CB05	11 ± 0.5 ^bcd^	31 ± 1.7 ^abc^	22 ± 2.9 ^bcd^	5.1	24.0
CB10	11 ±0.7 ^bcd^	25 ± 1.6 ^abc^	17 ± 1.5 ^cde^	5.4	27.0
CB30	11 ± 0.4 ^bcd^	33 ± 1.4 ^abc^	26 ± 3.1 ^ab^	6.0	31.5
CB60	11 ± 0.1 ^abc^	31 ± 2.2 ^a^	27 ± 1.6 ^a^	6.6	36.6
S-ext	12 ± 1.2 ^ab^	10 ± 1.0 ^f^	6 ± 1.8 ^gh^	1.8	7.0
SF05	10 ± 1.2 ^bcd^	11 ± 2.4 ^f^	6 ± 1.0 ^gh^	5.8	26.5
SF10	10 ± 0.3 ^bcd^	12 ± 0.0 ^f^	6 ± 1.2 ^gh^	5.3	25.8
SF30	10 ± 0.4 ^bcd^	30 ± 2.2 ^ab^	23 ± 1.6 ^abc^	6.7	31.1
SF60	10 ± 0.3 ^bcd^	32 ± 3.6 ^a^	25 ± 3.7 ^ab^	6.8	33.7
SB05	11 ± 0.3 ^bcd^	11 ± 3.5 ^f^	4 ± 0.7 ^h^	5.3	26.1
SB10	10 ± 0.5 ^bcd^	13 ± 0.6 ^ef^	5 ± 1.0 ^h^	6.2	30.0
SB30	10 ± 0.5 ^bcd^	22 ± 1.7 ^bcd^	13 ± 0.4 ^ef^	7.6	40.0
SB60	11 ± 0.5 ^bcd^	31 ± 3.3 ^ab^	22 ± 1.4 ^abc^	7.6	40.8

* Different letters indicate statistically different values (*p* ≤ 0.05) by one-way ANOVA and Tukey’s pairwise comparison. ** Measured based on single samples. Standard deviation was estimated to 0.5 g/L by Aspevik et al. [10].

## Data Availability

The data presented in this study are available in the article and the supplementary material.

## Supplementary Materials

The following are available online at https://www.mdpi.com/2304-8158/10/1/38/s1, Table S1: Chemical shifts of lactate methyl resonance.
